# Supplementary material for: Resident Satisfaction of Urban Green Spaces through the Lens of Landsenses Ecology
Source: Int J Environ Res Public Health. 2022 Nov 18;19(22):15242. doi: 10.3390/ijerph192215242 (PMC9690267; doi:10.3390/ijerph192215242)
Supplement: Supplementary file 1 [file ijerph-19-15242-s001.zip › ijerph-1980299-supplementary.pdf]

Supplementary Material

Resident's satisfaction of urban green spaces through the lens of Landsense ecology

Sinan He[1,2], Dingkai Chen[1], Xiaoqi Shang[1,2], Linwei Han[2,*], Longyu Shi[1]

[1] Key Laboratory of Urban Environment and Health, and Health Key Laboratory of Urban Metabolism of Xiamen, Institute of Urban Environment, Chinese Academy of Sciences, Xiamen 361021, China;

[2] University of Chinese Academy of Sciences, Beijing 100049, China

Corresponding Author:

Linwei Han

E-mail: hanlinwei@ucas.ac.cn

Tel. and Fax: +86-18813139622

Contents

Figure S1 Comparison of residents' perceptions in different neighborhoods.

Table S1 Socio-demographics and residential characteristics of the sample.

Table S2 Analysis of significant differences among respondents.

Table S3 Analysis of the rationality of conceptual model.

Table S4 Analysis of the convergent validity and discriminant validity.

Table S5 The path analysis of structural equation models.

Table S6 Test of mediating Effect.

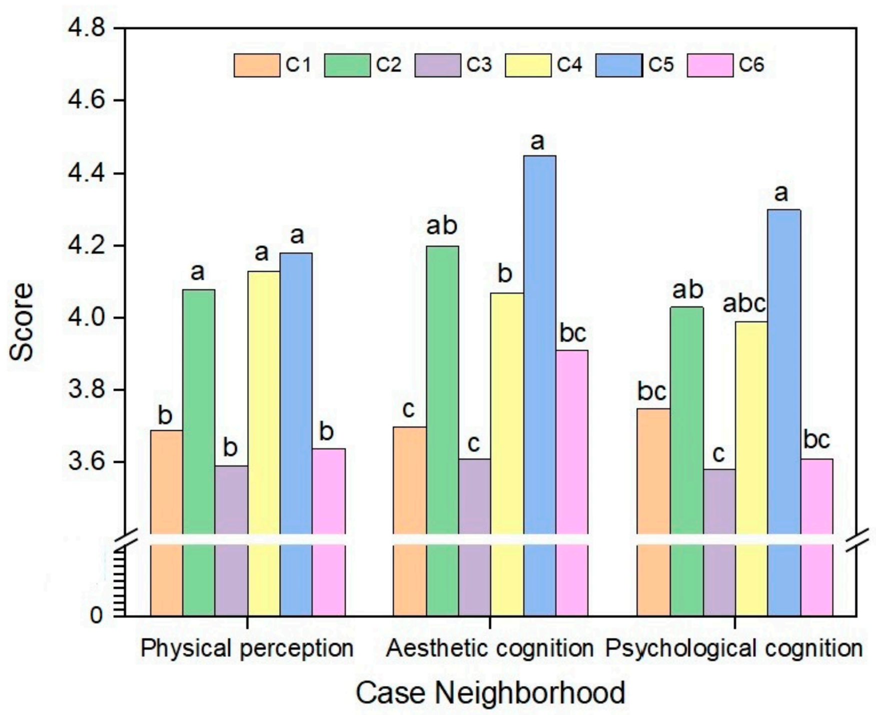

Figure S1 Comparison of residents' perceptions in different neighborhoods

Appendix.

Selection of observed variables

According to the conceptual model in Figure 1, physical perception (F1), aesthetic cognition (F2), psychological cognition (F3), and other perception (F4) are latent variables of our SEM. Quantifiable indicators as observed variables were selected for these latent variables. Observed variables of physical perception were selected in the aspects of vision, smell, audition, and touch. Taste was excluded because the ecological infrastructures in our case study mainly constituted of ornamental plants. Observed variables of aesthetic cognition were mainly selected from landscape aesthetic indicators. According to Tveit et al. (2006) [1], Wu et al. (2017) [2], Hur et al. (2010) [3], Frank et al. (2013) [4] and considering the features of urban residential ecological infrastructures. Landscape richness, spatial conformity, management status, and green coverage were selected as the observed variables of aesthetic cognition. The management status refers to the sense of order and the vestige of manual maintenance. Excessive management can induce a feeling of manual work, while insufficient management gives a feeling of dilapidation [5]. Observed variables of psychological cognition include safety, belonging, esteem, and willingness to communicate based on Maslow's Hierarchy of Needs. The observed variables are shown in Table 2.

Measurement of greenspace characteristics

(1) diversity of vegetation and landscapes

Shannon-Wiener Index was used to characterize the biological diversity of green space according to the following equation:

$$F = CR \times L \tag{1}$$

where F is the complexity of landscape components, CR is the number of landscape colors, and L is the number of landscape types.

(2) biomass of vegetation

Urban tree leaf area regression model was used to evaluate the biomass of vegetation [6]. The equation for calculating the tree leaf area was as follows:

$$Y = \exp(0.6031 \times 0.2375H + 0.6906D - 0.0123S) + 0.1824 \tag{2}$$

$$S = \pi \times D \times \frac{H + D}{2} \tag{3}$$

where Y is the total leaf area (m^2), H is the height of canopy (m), and D is the diameter of canopy (m).

For shrubs and grass, the leaf area was calculated using the formula as follows:

$$Y = LAI \times S \tag{4}$$

where Y is the total leaf area (m^2), LAI is leaf area index, and S is the plant coverage area (m^2).

(3) openness of landscapes

Building density and building space crowdedness was used to characterize landscape openness. Building density was calculated using the formula as follows:

$$C_b = \frac{\sum_{i=1}^{n} S_{bi}}{A} \times 100\% \tag{5}$$

where C_b is the building density, S_{bi} is the bottom area of the ith building (m^2), n is the total amount of building in the study area, and A is the total area of the study area (m^2).

Crowdedness of building space was calculated using the formula as follows:

$$SC_b = \frac{\sum_{i=1}^{n} V_{bi}}{\max\{H_b\} \times A} \times 100\% \tag{6}$$

where SC_b is the crowdedness of building space, V_{bi} is the volume of the ith building (m^3), n is the total amount of building in the study area, max{H_b}is the maximum height of the buildings (m), and A is the total area of the study area (m^2).

Table S1 Socio-demographics and residential characteristics of the sample

Factors	Categories	Proportion
Gender	Male	0.46
	Female	0.54
Age	11 - 20	0.09
	21 - 30	0.20
	31 - 40	0.30
	41 - 50	0.12
	51 - 60	0.16
	61 and above	0.13
Place of Birth	Xiamen City, Fujian Province	0.29
	Other City, Fujian Province	0.41
	Other Province	0.31
Education	Junior high school and beneath	0.22
	Senior high school	0.14
	College	0.16
	Bachelor	0.43
	Master or above	0.06
Length of residence	Less than 1 year	0.03
	1-3 years	0.46
	4-6 years	0.37
	6-9 years	0.07
	9-12 years	0.06
	More than 12 years	0.00
Monthly household income	Less than ￥5000	0.08
	￥5000～ ￥10000	0.17
	￥10000～ ￥20000	0.39
	￥20000～ ￥30000	0.21
	￥30000～ ￥50000	0.08
	￥50000～ ￥100000	0.03
	>￥100000	0.04
Residence	Own property	0.81
	Rented	0.16
	Dormitory or other	0.03
Marital status	Single	0.14
	Dating	0.03
	Married	0.83

Occupation	Employee of an enterprise	0.26
	Waiter/waitress	0.04
	Student	0.10
	Entrepreneur	0.06
	Employee of a public institution	0.15
	Researcher	0.01
	Freelancer	0.12
	None	0.07
	Retired	0.19

Table S2 Analysis of significant differences among respondents

Variables	Count	H	SD	p-Value
Visual field (I1)	399	22.53	5	***
Temperature amenity (I2)	399	10.29	5	0.07
Humidity amenity (I3)	399	11.52	5	0.04
Air freshness (I4)	399	20.89	5	0.001
Plant odor (I5)	399	20.95	5	0.001
Noise (I6)	399	38.78	5	***
Landscape richness (I7)	399	64.56	5	***
Spatial conformity (I8)	399	42.37	5	***
Management status (I9)	399	30.71	5	***
Green coverage (I10)	399	55.52	5	***
Safety (I11)	399	43.49	5	***
Belonging (I12)	399	28.88	5	***
Esteem (I13)	399	42.88	5	***
Willingness to communicate (I14)	399	30.33	5	***
Housing quality (I15)	399	9.74	5	0.08
Transport convenience (I16)	399	68.18	5	***
Community service (I17)	399	17.54	5	0.001
Recreation facility (I18)	399	19.7	5	0.001

*** p-value lower than 0.001

Table S3 Analysis of the rationality of conceptual model

Variables	Dimension			
	1	2	3	4
Belonging (I12)	0.76	0.20	*	0.25
Safety (I11)	0.76	0.22	0.14	0.20
Esteem (I13)	0.73	0.29	0.12	0.24
Willingness to communicate (I14)	0.67	0.20	0.28	*
Spatial conformity (I8)	0.18	0.77	*	0.14
Landscape richness (I7)	0.22	0.77	0.10	0.13
Management status (I9)	0.16	0.73	0.22	0.23
Green coverage (I10)	0.32	0.61	*	0.13
Community service (I17)	0.23	0.24	0.72	0.16
Transport convenience (I16)	*	*	0.70	0.11
Recreation facility (I18)	0.18	0.24	0.67	0.11
Housing quality (I15)	0.32	0.12	0.58	0.21
Temperature amenity (I2)	0.18	0.18	0.16	0.83
Humidity amenity (I3)	0.13	0.18	0.24	0.79
Air freshness (I4)	0.36	0.22	0.13	0.59

* p-value lower than 0.10

Table S4 Analysis of the convergent validity and discriminant validity

Constructs	Convergent Validity		Discriminant Validity			
	CR	AVE	F1	F2	F3	F4
F1	0.77	0.53	**0.73**			
F2	0.74	0.49	0.61**	**0.7**		
F3	0.83	0.54	0.65**	0.70**	**0.74**	
F4	0.67	0.47	0.64**	0.61**	0.65**	**0.68**

** p-value lower than 0.01

Table S5 The path analysis of structural equation models

DV	IV	Std. Est	S.E.	Est./S.E.	P-Value	R^2
F3	F1	0.29	0.08	3.43	0.001	0.54
	F2	0.43	0.08	5.55	***	
	F4	0.25	0.07	3.79	***	
I19	F1	-0.01	0.05	-0.11	0.91	
	F2	0.33	0.06	5.69	***	
	F3	0.52	0.06	9.10	***	

*** p-value < 0.001

Table S6 Test of mediating Effect

Path	Product of Coefficients	Estimate	S.E.	Est./S.E.	P-Value
F1→F3→I19	a1*b3	0.24	0.06	4.07	***
F2→F3→I19	a2*b3	0.35	0.11	3.26	0.001
F4→F3→I19	a3*b3	0.17	0.05	3.44	0.001

*** p-value < 0.001

References

1. Tveit, M.; Ode, Å.; Fry, G. Key concepts in a framework for analysing visual landscape character. Landsc. Res. 2006, 31, 229–255. ttps://doi.org/10.1080/01426390600783269.
2. Wu, J.S.; Yuan, T.; Wang, T. Preliminary theory of urban landscape esthetics based on three-dimensional landscape indicators. Acta Ecol. Sin. 2017, 37, 4519–4528. https://doi.org/1000-0933(2017)37:13<4519:JYSWJG>2.0.TX;2-Z.
3. Hur, M.; Nasar, J.L.; Chun, B. Neighborhood satisfaction, physical and perceived naturalness and openness. J. Environ. Psychol. 2010, 30, 52–59. https://doi.org/10.1016/j.jenvp.2009.05.005.
4. Frank, S.; Fuerst, C.; Koschke, L.; Witt, A.; Makeschin, F. Assessment of landscape aesthetics-Validation of a landscape metrics-based assessment by visual estimation of the scenic beauty. Ecol. Indic. 2013, 32, 222–231. https://doi.org/10.1016/j.ecolind.2013.03.026.
5. Coeterier, J.F. Dominant attributes in the perception and evaluation of the Dutch landscape. Landsc. Urban Plan. 1996, 34, 27–44. https://doi.org/10.1016/0169-2046(95)00204-9.
6. Nowak, D.J.; Crane, D.E. The Urban Forest Effects (UFORE) Model: Quantifying Urban Forest Structure and Function; U.S. Department of Agriculture, Forest Service, North Central Forest Experiment Station: St. Paul, MN, USA, 2000.
